# An American Text-Book of Physiology

**Published:** 1901-04

**Authors:** 


An American Text-Book of Physiology. Edited by William H. Howell, Ph. D., M. D., Professor of Physiology in the Johns Hopkins University. Second edition, revised. Vol. II. Philadelphia and London: W. B. Saunders & Co. 1901. [Price, cloth, $6.00 net; sheep or half morocco, $7.00, net.]

The contributors to Volume II. of the American Text-book of Physiology are Professors Bowditch, of Harvard; Donaldson, of Chicago; Lee, of Columbia; Lombard, of Michigan, and Sewell, of Denver. The same careful revision that marked the first volume is evident here. The section devoted to the nervous system has been entirely rewritten and is the most interesting, as it is the most important in the book. The other sections take up the general physiology of muscle and nerve, the special senses, special muscular mechanisms, and reproduction. Again the publishers are to be commended for their wisdom in replacing the single volume by two.

